# To Talk or not to Talk About Existential Questions – An Interview Study With Elderly Persons and Patients With Fatal Disease

**DOI:** 10.1177/10499091241276862

**Published:** 2024-08-21

**Authors:** Carl Bäckersten, Stina Nyblom, Ulla Molander, Inger Benkel

**Affiliations:** 1 Region Västra Götaland, Sahlgrenska University Hospital, Palliative Centre, Gothenburg, Sweden; 2 Institute of Medicine, Sahlgrenska Academy, 3570 University of Gothenburg, Gothenburg, Sweden

**Keywords:** existential questions, existential care, spiritual, relationship, end-of-life, meaning of life, existential conversations, existential dimension

## Abstract

**Background:** Existential experiences often come to the fore in the case of a severe and/or life-threatening disease and in old age. This can evoke a variety of thoughts and emotions. The existential dimension is a concept that encompasses spiritual, religious and secular perspectives. **Objective:** The aim of this study was to gain a deeper understanding of how patients describe the existential dimension of life and whether and in what way the existential questions are raised in conversations and other forms of support within care. **Methods:** A qualitative design, with in-depth semi-structured interviews with patients admitted to an out-clinic oncology department at one hospital and participants living in a nursing home. The interviews were analysed using qualitative content analysis. **Results:** A total of 15 persons were interviewed. The respondents varied in age from 44 to 96. Two main themes emerged: What are existential questions? and Talk about existential questions. The existential questions refer to life as a whole and death as an end of life. It was summarized into three subcategories: *The experience of the life, Existing within context *and* Spirituality and religion*. About half of the participants thought it was important to talk about existential questions. They wanted to choose who to talk to, when and about what. Support was received from loved ones, professionals and experiences beyond conversations. **Conclusions:** The study provide new knowledge of how patients and elderly experience the existential dimension. The respondents emphasized a desire to be selective with whom they shared these questions and thoughts.

## Introduction

Existential questions derive largely from philosophers (eg, Kierkegaard, Nietzche and Heidegger) who have contributed to a shared line of thought over centuries. The existential philosophical tradition includes both theistic and secular world views, and it has been argued that fundamental values and beliefs should have a common label.^1^ Central existential issues are meaning and loss of meaning in life which is important in discussions around existential issues.^2,3^

Existential experiences often come to the fore in the case of a severe and/or life-threatening disease and in old age. This can evoke a variety of thoughts and emotions, such as anxiety about death, hope and hopelessness, meaning and loss of freedom, which are linked to relationships, social connections, isolation and loneliness.^4-8^ Historically, and especially in recent years, there has been a debate about which conceptual apparatus is best suited to encompass these issues in care. Some persist with the concept of spiritual care, while more and more highlight ‘existential’ as a more suitable overarching concept.^9^

In palliative care, the inclusion of existential/spiritual dimensions is considered important to achieve holistic care. The European Association for Palliative Care (EAPC) reference group for spiritual care has addressed this question and provides the following definition of ‘spirituality’: Spirituality is the dynamic dimension of human life that relates to the way persons (individual and community) experience, express and/or seek meaning, purpose and transcendence, and the way they connect to the moment, to self, to others, to nature, to the significant and/or the sacred.^10^

The Scandinavian countries are often regarded as highly secular societies, but, in accordance with the post-secular perspective, this does not mean an absence of spirituality and religiosity. According to Nissen et al (2021),^11^ spiritual care can be understood as trying to comprehend a patient’s existential orientation, needs, and resources in religious, spiritual, and secular domains. These three domains can all be considered 'existential' and are embedded within a cultural context and influenced by, for example, family, social, historic, economic and educational aspects. As mentioned above, ‘existential’ can thus be considered the most open and best-applicable concept in this context.^12,13^

In the literature, ‘spiritual’ is the term most commonly associated with spiritual and existential issues. However, ‘existential’ can be seen as a broad term for issues related to people’s experiences and ways of thinking about life; in Sweden it is widely used, and often considered to be the most suitable term, when referencing these matters in a health care context.^12,14,15^

To the best of our knowledge, there are limited studies asking patients with fatal disease and elderly directly about their perception of the existential dimension. The aim of this study was to gain a deeper understanding of how the elderly and patients with fatal disease perceive and describe the existential dimension of life in face-to-face interviews, and whether, and in what way, the existential questions are raised in conversations and other forms of support within care.

The research questions were: How do you describe existential questions? Is it important to be able to talk about existential thoughts? Do you talk to anyone about these issues and, if so, who? What support do you need in these matters? Can healthcare professionals help you with these questions?

## Methods

### Design

A qualitative design was chosen, underpinned by a hermeneutic approach that sought to discover participants’ experiences to gain a deeper understanding of the existential questions and thoughts. In the hermeneutic position, researchers aim to make sense of how the participants reflect on their own experiences, while simultaneously examining how the researchers’ views and biases might impact the analytical process.^16^ This study was approved by the Swedish Ethical Review Authority (No. 2021-00956; date of approval: 17/03/2021).

### Participants and Settings

Patients with a fatal cancer disease at an outpatient oncology clinic connected to a city hospital, and elderly people living in a nursing home, in an urban part of western Sweden were considered for inclusion. The elderly could no longer fend themselves in their own home and needed help around the clock.

The Swedish word for ‘existential’ was used in the interviews, encompassing spiritual, religious and secular issues of an existential nature. The deliberate choice of this word is linked to previous reasoning about the concept’s meaning and that the concept existential is known and used by professionals.^12-15^ To achieve the objective of the study, questions were asked about existential issues, needs and care. The inclusion criteria were that participants had to be 18 years or older, understand and speak Swedish, and have the capacity to provide informed consent.

### Data Collection

Data were collected over a 7-month period between April 2022 and October 2022. Information about the study was provided via a brochure that was available in the waiting room at the outpatient clinic where the participants were being treated. At the nursing home for the elderly, a nurse provided information to persons who met the inclusion criteria.

After being informed about the study, those willing to participate sent a signed consent form in a prepaid envelope to the researcher, who contacted the participants by telephone and arranged interviews with them. Only one interview was offered due to the vulnerable situation of the participants with progressive disease and/or old age.

In total, the outpatient clinic and the nursing home received 70 brochures, of which 35 reached patients/elderly. Of the 15 persons who gave their consent, all participated in the study. In-depth semi-structured interviews were conducted on a face-to-face basis.

The interviews were arranged according to the wishes of the participants and were conducted either at the participant’s home, at the outpatient clinic, digitally, at the nursing home or via phone call. The interviews were digitally recorded and professionally transcribed verbatim. Transcripts were not returned to participants for comment and/or correction, due to ethical reasons based on their vulnerable situation. An interview guide was used containing the questions mentioned in the introduction. The interview guide was based on a previous survey conducted with professionals where the questions were tested and modified to be as understandable as possible. In order to capture the respondents’ initial spontaneous thoughts about existential questions, the first question (How do you describe existential questions?) was posed without further explanation, and the participants were given time to ponder their answer. The interviewer could then ask follow-up questions to stimulate conversation.

The interviews were performed by social workers trained in interview techniques, but none were involved in caring for any of the participants they interviewed. The duration of the interviews ranged from 10 to 40 minutes. The length of the interviews was controlled by the participants' ability to participate and what they wanted to talk about.

### Data Analysis

Transcribed interviews were analysed using qualitative content analysis to identify both manifest and latent meaning.^17,18^ All the authors individually marked text relevant to the research questions, which was later confirmed in a joint discussion. Through further analysis, units of meaning were identified and grouped into themes and subthemes. The most common findings were described and compared against each other, and supporting quotes were linked. A joint analysis was performed by all the authors to interpret the results as objectively as possible. Any discrepancies were discussed until a common conclusion was reached.

## Results

A total of 15 persons were interviewed. The respondents varied in age from 44 to 96 years and the majority were women. For more details, see Table 1.

**Table 1.** Demographic Data.

Participants (n = 15)	
Gender	
Female	14
Male	1
Age (year)	
40-50	1
51-60	2
61-70	3
71-80	2
81-90	5
91 ---	2
Living situation	
Alone	11
Cohabiting	4
Employment status	
Employed	4
Retired	11
Highest level of education	
Primary school	2
High school	5
University	8
Participant’s disease/condition	
Heart disease	1
Cancer	8
Old age	6

In the interviews, two main themes emerged: What are existential questions? and Talk about existential questions. This study asked patients (P) and elderly (E) about their own views on existential questions. To illustrate the participants’ perspectives quotations expressed by participants in the interviews are presented below each heading.

### What are Existential Questions?

When asked what existential questions are, participants rarely gave a straight answer, but rather engaged in an extended line of reasoning. Some initially said they did not understand the word and others found it a very broad question and did not know where to start. However, after reasoning with themselves and arriving at an answer to the question that they felt was their position, the majority identified an answer that tended to refer to life as a whole.

One participant described existential questions as being about “My moment on earth, what I prioritize and how I live my life”(2,P), and another expressed that they are about “What life can mean in general”(3,P).

Death was also presented in the existential questions as an end to life, *“Existence has a beginning and an end. Then it’s charged because it’s about my existence, because this is my moment on Earth that has an end”(2,P)*, although for some participants it meant a transition to something beyond death.

In participants’ reasoning around the existential questions, several aspects emerged that could be summarized into three subcategories: The experience of life, Existing within a context, and Spirituality and religion.

### The experience of life

One aspect within participants’ existential thoughts was the life lived: *“What you do with your life and how to develop yourself to take care of these (existential) issues”(8,E)*. It was often through their narrative life story that many conveyed their views on what has been existentially important in life and how they currently experienced life. This sometimes included practical examples, such as how to raise children and how to transfer one’s values to family and loved ones. Sometimes it was about choices in education and professional life as well as how to find meaning in life. *“I want to be kind*. *I want to be considerate*. *I want to do a good job*. *I want to be open in my communication and I want to have a positive view of myself and other”(9,P)*.

### Existing within a Context

Another recurring topic was relationships and the importance of existing within a context which was described by the participants as identifying with having a relationship with someone else where you are important to each other. *“To be human is to be in a relationship with others, children have meant a lot to me.. and I’ve had two sisters that I’ve had to take care of.”(5,E)*.

The relationships participants referred to were often those in their private lives, such as with family and friends, but some also mentioned relationships developed at work or through leisure interests, such as within a congregation or association.

Relationships were seen as key to existing within a context, and having few relationships could mean losing a sense of coherence. Becoming seriously ill or growing old could be reasons for changing life circumstances. Some who had become seriously ill had found that people around them could become reserved and withdrawn. In some cases, participants could only manage to maintain contact with a few people, which sometimes led to their relationships with others diminishing.

For the elderly participants, it was sometimes the case that those close to them had fallen ill and died, or that they themselves could no longer maintain previous relationships.

“You lose your beloved husband, you lose your relatives, you lose old good friends. All the time you have to deal with questions about how to cope with life after you have experienced so many losses”(5,E).

### Spirituality and Religion

Faith, spirituality and religion were described by many participants as part of the existential dimension of their lives. Some expressed having faith, while others distanced themselves from religion and other beliefs. In some instances, religion served as a framework allowing participants to process existential questions, such as how one should live their life, and provided guidelines for their own outlook on life. As one participant said *“I have been to church and learned a lot there, about how to live, how to do it best and how you can develop*. *that you are interested in learning different things in life*.*”(8,E)*. Those who did not cite religion described how they tried to find other ways to deal with existential questions. *“If you don’t have belief, you have to find your own way”(2,P)*.

### Talk About Existential Questions

About half of the participants thought it was important to talk about existential questions and thoughts. There was a wide range of responses in this regard, with some participants saying that it was not important at all while others considered it very important. This polarization is illustrated by the following quotes: *“But why is it so important to talk about it then? If you have a need to talk about anything, then you do”(1,P)* and *“It is very important to me* … *These are probably the biggest questions we face, why do we exist, here and now, on earth”(9,P)*.

Some participants described a need to discuss their thoughts with someone else. Participants were selective about who they wanted to talk to and pointed out the need to have confidence that the person was wise and trustworthy. “You shouldn’t push existential questions on people when they don’t have any. You can just offer them and ask how are you, so if you can talk about it, you get it.. I think it's important that the opportunity exists, but as I said, not everyone wants to talk about it anyway..”(5,E).

Some felt that young people did not have the same life experiences, which made it more difficult to talk to them. Most stated that they talked to persons amongst their network of family and friends. But the choices of confidants varied, depending on the nature of the relationship. *“You also choose who to talk to out of consideration, and because I know that my children are different my son chose not to ask much… he didn't want to know more*.. *but my daughter she could take it*.”*(2,P)*.

Some stated that it was sometimes easier to talk to professionals, such as physicians, registered nurses, social workers, priests, etc. One participant expressed that it was about courage and that professionals needed to be brave enough to face a person’s existential questions: *“There are situations in which you also have to be brave as a healthcare professional to raise certain existential questions”(2,P)*.

The participants gave a number of details of the content of their conversations, from practical considerations such as funerals, wills and medical issues to the need to discuss questions about life and its meaning. The variation in content is shown by the following quotations: *“That you have arranged how you want it, in which church and cemetery and all that and talk about how you want it to be in late life ... how to live and how it should be”(7,E), “One thing that can break a vicious circle is that you get medicine”(5,E)*.

Some described conversation as a source of support with regard to existential questions, and mentioned certain factors as being important, such as someone giving their time, instilling trust, attitude and continuity over time. *“I think this health care thing is actually interesting because it’s getting a lot of people who have never thought about existential questions to do s*o. *I think it could be very helpful for many people to have the opportunity to at least talk to someone about this”(9,P)*.

Beyond conversation, some participants described other experiences that they perceived as examples of support. These included going to a concert, experiencing nature, participating in activities or being with others. “I think everyone needs some kind of support ... go out and meet people, join an activity, go to yoga. How much it gives.. Because it's easily done.. you feel bad or you don't feel well and then you don't get out and then it just gets heavier and heavier.”(3,P).

For those who practised religion, the availability of religious representatives was seen as a form of support, both for conversation and practising religious rites.

## Discussion

This study aimed to explore how patients with a fatal disease and elderly persons describe their own views on existential questions.

While some of the participants gave a direct answer, others hesitated and initially struggled to define the concept of existential questions, needing to reason their way to an answer. There was, however, a consensus among the participants that existential questions were about what it is like to be human. The participants’ further reasoning about existential questions aligned closely with established philosophers, for example Frankl, as well as with previous studies.^2,19^

Many of the participants recounted a narrative from their life, the content of which reflected their existential thoughts. Through their narrative life stories, they could share their views on what is existentially important in life and reflect on the life they had lived. The narrative life story as a phenomenon is recognized in palliative care and in person-centred care. A narrative life story, according to Coleman (1999),^20^ should reflect someone’s experiences and allow them to come to terms with their own history and what has been important to them in life.

A prominent topic highlighted in the study was the importance of existing within a context, related to relationships to other persons, different forms of networks or as a part of a congregation. Both illness and old age can diminish this and cause existential loneliness, as previously described.^4,5^

Northern European countries are seen as distinctly secular. Hvidt et al (2022)^9^ describe “the existential” as a useful overarching concept with secular, spiritual and religious domains. In this study, based in a secular context, religion, faith and spirituality were also highlighted as part of participants’ existential experiences. This was seen regardless of the participant’s age or the nature of their illness.

Conversation is often highlighted as the most common source of support with regard to existential issues. However, in this study it emerged that only half of the participants felt the need to talk to others about these issues. Of those who wanted to talk to someone, many stated that their own network was a sufficient resource for them.

How one communicates in one’s private network is influenced by communication patterns and what is perceived to be an acceptable discussion. In this study, the participants highlighted the need for selectivity when it came to choosing someone to confide in, in line with previous studies with reports from both patients and professionals.^21,22^

Some participants expressed that professionals should be available for discussions around existential issues, when the patient/elderly felt the need for it. These discussions could be practical in nature, such as arranging a religious ritual or funeral, or include issues related to care and nursing, which the participants linked to existential needs as reported by professionals.^23^

Participants also described professionals as interlocutors, specifically mentioning social workers, psychologists, priests, and deacons.

It was highlighted that good conversations depend on there being an interlocutor in whom they can trust and who has time to listen without feeling stressed.

According to the participants, it is important that professionals have the courage to face existential questions, are open and invite people to discuss issues that are important to them, identified in studies of medical records^5^ and professionals.^19,24^

Regardless of whether the need for conversation concerned practical issues, rituals or reflective conversations, the participants described having the opportunity to satisfy these needs mainly within their own network. If needed, professionals could also provide support. The participants stated that the choice of interlocutor should be their own. They themselves wanted to own the question of whether or not to talk about existential issues, and not for someone else to decide.

It was not just conversations that were perceived as a source of support for existential needs. Various forms of activities and experiences were described as providing relief for existential distress, which could be just as important to the individual as a conversation.^5^

With the deliberate choice of the word “existential” in the initial interview questions, the study provides some insight into the practical usefulness of the concept in conversations with patients and elderly. Although most participants were able to express their thoughts around the concept, the results show that some participants seemed to have difficulty defining its meaning. The question remains whether this is due to the specific group of participants in this study, the word/concept being too academic, or alternatively that it is not the word that is difficult but the topic itself, which could be subject for further research.

## Conclusion

This study included participants of different ages and with different diseases, the majority of whom grasped the term “existential” and provided a unified and coherent description of the existential dimensions of their lives.

A clear trend emerged, with participants associating existential questions with the life they had lived and their life as it was currently, as well as with death as a part of life. The respondents emphasized a desire to be selective with whom they shared these questions and thoughts. Those who experienced a need for support often received it from within their own network, but in certain situations professionals could fulfil an important function. Support was described in the form of conversations, but other activities, both secular and religious, were also perceived as beneficial. This first-hand report from elderly and patients with fatal disease contributes to the understanding of the perception of the existential dimension among vulnerable persons, which can be valuable for healthcare professionals dealing with existential issues.

## Strengths and Limitations

The study’s choice of the existential concept use can be considered both as a limitation, as the results show that some participants had difficulties understanding its meaning, and a strength, as the study has examined the concept in practice. Another limitation is that the survey language was Swedish, which excluded non-Swedish-speaking people who may have given different perspectives on the research questions. Males were also underrepresented. Swedish society is regarded as highly secular, which could limit transferability outside Scandinavia. Regardless of age, illness and residence, there was consensus in how existential questions were perceived, which can be seen as a strength, despite the study’s small sample size.
